# Climate-mediated population dynamics for the world’s most endangered sea turtle species

**DOI:** 10.1038/s41598-023-41647-8

**Published:** 2023-09-02

**Authors:** Michael D. Arendt, Jeffrey A. Schwenter, David W. Owens

**Affiliations:** 1 South Carolina Department of Natural Resources, Marine Resources Division, 217 Fort Johnson Road, Charleston, SC 29412 USA; 2 College of Charleston, Grice Marine Biology Laboratory (Retired), 205 Fort Johnson Road, Charleston, SC 29412 USA

**Keywords:** Ecology, Climate-change ecology, Climate and Earth system modelling, Herpetology

## Abstract

Restricted range, and subsequently small population size, render Kemp’s ridley sea turtles (*Lepidochelys kempii*) the most globally endangered sea turtle species. For at least two decades preceding conservation, high egg harvest rates reduced annual cohort recruitment. Despite > 50 years of dedicated conservation, annual nest counts remain well below a landmark 1947 level. Prior studies attribute less robust than anticipated nest count rebound to multiple contemporary concerns; however, analyses herein convey optimistic interpretation. In objective 1, improved analysis of the ratio of hatchlings to nests since 1966 suggested age structure stabilization as a more likely basis for nest count trends after 2005 than density-dependent effects. In objective 2, multiple regression revealed a lagged (≤ 13 years prior) climate influence on nests (adj. r^2^ = 0.82) and hatchlings per nest (adj. r^2^ = 0.94) during 2006–2022. In objectives 3 and 4, a simulator modeled population response to changes in a suite of demographic rates including survival. Across 32 models, high survival and dynamic cohort sex ratio, sexual maturity age, and the ratio of clutch frequency to remigration interval best explained nesting trends during 1966–2022. These novel findings provide alternative perspective for evaluating species recovery criteria and in turn refine future nest trend expectations.

## Introduction

In wildlife management, balancing protections with optimizing resource use remains existential.

For the past six decades, human perceptions of global sea turtle species have mostly shifted from a commodity for consumption^1^ to a flagship conservation species with economic value^2^. Legislative acts initially protected sea turtles on nesting beaches but also prohibited their trade. In-water protection began in the 1990’s following the deduction that drowning in shrimp trawls posed the greatest anthropogenic threat to sea turtles residing in nearshore coastal waters of the Southeast United States^3^. Other trawl fisheries later adopted similar in-water protective measurements^4^ and reducing incidental capture in other fisheries remains a global priority^5^. Concurrent with decades of conservation, nest counts for green sea turtles (*Chelonia mydas*) in the Pacific Ocean^6^ and Caribbean^7^ and loggerhead sea turtles (*Caretta caretta*) in the Northwest Atlantic Ocean^8^ now exhibit stable to increasing trends.

In contrast to green and loggerhead sea turtles in the Americas since the 1990s, managers exercise a more cautious interpretation of annual nest count data^9^ for the Kemp’s ridley sea turtle (*Lepidochelys kempii* [Garman 1880]). Most annual nesting for this species occurs in the western Gulf of Mexico (GOM) in the state of Tamaulipas, Mexico^9^. The oldest quantified nesting reference in Tamaulipas, Mexico stems from an “arribada” nesting event on 18 June 1947^10^. Initially estimated to comprise 40,000 nesters^10^, subsequent investigations later suggested the 1947 arribada comprised between 6,000^11^ and nearly 30,000 nesters^12^. Recent re-analysis of video data from the 1947 arribada also suggests that Kemp’s ridley sea turtles laid between 82,514 and 209,953 nests in 1947^12^. Despite conservation in this region since 1966, through 2022, nest counts remain ≤ 23%^9^ of the lower 1947 estimate^10^. Given that Kemp’s ridley sea turtles typically achieve sexual maturity in their first decade^13^, researchers anticipated a far more robust increase in annual nesting than what transpired^14,15^.

A recent stock assessment for Kemp’s ridley sea turtles^14^ reported strong influence of anthropogenic mortality sources on nesting, but further scrutiny revealed methodology concern. First, size/age structure of stranded sea turtles traditionally derives modeled survival rates^9^ but variable cohort recruitment and/or demographic changes also influence observed structure^16^. Second, modeling survival rates without first parameterizing rates for a null model devoid of change in age structure or annual nest counts encourages statistical artefacts. Retrospective analysis of parameterizations used by prior Kemp’s ridley sea turtle modeling studies revealed unintentional but inherent bias for population growth (see Supplement S1). Consequently, failure to achieve inflated modeled nest count necessitated the conclusion of ‘reduced survival’ to account for discrepancies between observed vs. predicted nest counts. Third, some studies suggest increased survival in recent decades^15^, consistent with low (≤ 2%) incidental capture frequency in the commercial shrimp fishery since 2011^17^. Increased contemporary survival could exacerbate abundance disparity between mature females and mixed-sex juveniles^18^.

Dynamic age structure^19^ may also reflect systemic rather than erratic change^18–20^; thus, static parameterization of demographic rates represents a second shortcoming of prior modeling efforts for Kemp’s ridley sea turtles^19^. Modeling multiple static maturation ages^14,15,21,22^ brackets a grand nest count range but inhibits consideration of an oscillatory nest count pathway for regulating population density. Temporal variability in clutch frequency and/or remigration interval may explain contemporary nest count variability^23^ but the mechanisms underlying such variability remain poorly documented. Studies report climate influence on annual nest counts for both loggerhead^24–26^ and green^27^ sea turtles in the region, but analyses of Kemp’s ridley sea turtle data in the context of climate remain sparse. Strong winds reportedly influence arribada phenology and may facilitate the return of nesters from the beach to the sea plus cover their egress tracks^10^. Particle simulations demonstrate inverse hatchling dispersal success from Tamaulipas, Mexico with cyclonic activity^28^. Climate indices also predict^16^ particle emigration from the GOM^29^, which has implications for estimating the abundance of Kemp’s ridley sea turtles in the GOM^30^.

Given climate influences reported for some aspects of Kemp’s ridley sea turtle life history, this study sought to better understand the potential role of climate on annual nesting trends. Time series analysis (Objective 1) and multiple regression (Objective 2) evaluated the relative impacts of density-dependence^18^, changes in reproductive frequency^23^, climate association, and assemblage rebuilding on nesting trends since 1966. For the third objective, a population simulator established a “robust modeling framework^18^” to assess the relative importance of a suite of life history variables on nesting trends. For objective four, simulated historical perturbations downsized the modeled assemblage to the suspected abundance level in 1966. Subsequent characterization of demographic structure and exploration of nesting associations conformed with the call for sea turtle researchers to integrate abundance and demography^31^.

## Methods

### Data sources

Tamaulipas, Mexico (23.2°N) denotes the epicenter of Kemp’s ridley sea turtle nesting^15,20^. Beach patrols began in 1966 and the National Commission of Protected Natural Areas—Mexico now manages data collection. Data comprised annual counts of nests and hatchlings that for analysis standardization^9,14,15,21,22^ emphasized three adjoining beach segments: Rancho Nuevo, Tepehuajes, and Barra del Tordo/Playa Dos. Data acquisition occurred as follows:

(i) previously compiled, 1966 through 2014^9^; (ii) online annual reports, 2015 through 2020; https://www.gulfspillrestoration.noaa.gov/project?id=62, accessed 13 April 2023); or (iii) annual reports provided by affiliated program personnel, ≥ 2021.

Data for six climate indices (compiled prior to 1 June 2023) enabled association evaluation with nesting data: (a) unsmoothed, long-format Atlantic Multidecadal Oscillation (AMO), https://psl.noaa.gov/data/correlation/amon.us.long.data); (b) Atlantic Meridional Mode, sea surface temperature and wind, https://psl.noaa.gov/data/timeseries/monthly/AMM/); (c) North Atlantic Oscillation, www.cpc.ncep.noaa.gov/products/precip/CWlink/pna/nao.shtml); (d) Atlantic hurricanes (https://www.nhc.noaa.gov/data/#hurdat); (e) El Niño-Southern Oscillation, www.cpc.ncep.noaa.gov/products/analysis_monitoring/ensostuff/ensoyears.shtml.

Data sets began in 1851 (Atlantic hurricanes) to 1856 (AMO), or between 1948 and 1950. Hurricane data consisted of a maximum of four daily observations with associated geographical position and maximum wind speed data. Remaining climate data sets comprised monthly or running tri-monthly (El Niño-Southern Oscillation) values.

Data management and analysis benefited from two software packages. Microsoft Excel (v2016, Microsoft Corporation, Redmond, CA) supported data management, plotting, population simulation, and descriptive analysis. Minitab (v21, Minitab Corporation, State College, PA) supported multiple linear regression, normality testing (Anderson–Darling), cluster analysis, and significance (α = 0.05) testing (Pearson correlation, Kruskal Wallis analysis of variance).

### Objective 1: time series analysis of nesting data

Nest counts reflect the sum of clutches laid by reproductively active females each year, but in turn mask the proportionate contribution from each component. Conversely, when expressed as the absolute magnitude of proportionate change [abs{(Nest_*t*_ – Nest_t*-1*_)/ Nest_t*-1*_}], inter-annual nest count change permits general trend assessment. Specifically, no temporal trend indicates offsetting changes between female abundance and their reproductive activity. Alternatively, temporal increase(decrease) in absolute proportionate inter-annual magnitude indicates abundance increase(decrease) and/or reproductive activity decrease(increase). As such, analysis of the absolute proportionate inter-annual nest count change between 1966–1985, 1986–2005, and 2006–2022 permitted broad assessment of temporal changes in nest count components.

Evaluating inter-annual differences as a cumulative series also has statistical merit^32^; thus, summing proportionate inter-annual nest count variability created a cumulative distribution from 1966 (0) through 2022. A series of modified sine waves, computed by dividing each y-axis value by a constant ranging from 0.25 to 15 at 0.25 increments, contextualized periodicity during 1966–2022. The smallest constant to achieve a correlation of ≥ 0.99 with the cumulative distribution then selected the best periodicity. Multiplying each y-axis value of the selected series by half the range of the cumulative series appropriately scaled the series amplitude.

Life history variables form a feedback loop equation; thus, temporal variability in ratios computed among such variables also provide important insight regarding population trajectory. Prior analysis of a ratio of hatchlings to nests suggested density-dependent mediation of annual nesting for this species^20^; however, the previous metric masked several statistical concerns. First, prior analysis disproportionately scaled hatchlings (i.e., cumulatively) to nests (i.e., discrete annual counts); thus, inherent bias forced density limitation. Females nest more than once in their lifetime, so summing nests seems sensible. Second, following extensive egg poaching^10^, temporal autocorrelation existed between potential age structure rebuilding due to conservation and increasing mature female abundance. Therefore, in the present study a 10-year lagged ratio^20^ of cumulative nests to cumulative hatchlings re-evaluated the suggestion of density-dependence. Lastly, barring life history changes, population stability occurs when annual hatchlings per nest support the equivalent future nest count. As such, in the present study, cumulative hatchlings per nest, reported both independently and as deviation from an asymptotic or maximum value, provided a proxy temporal indicator of life history stability.

### Objective 2: climate prediction of nesting metrics

Multiple linear regression with climate data inputs generated predictive equations for annual nest counts and hatchlings per nest. Source data constrained to 2006–2022 minimized historical perturbation impacts on assemblage structure. For each nesting metric, the lowest relative Mallows Cp score selected the best regression model configuration^33^. Prior to regression, linear correlation selected a single best climate time series lag (i.e., year of to 16 years prior) relative to nesting metric year. In addition to 17 lag intervals, disparity between annual nesting but sub-annual climate data supported pre-regression data selection^34^. Hurricane tracks also reflected sub-annual partitioning of a 9-cell grid across three latitude (< 18, 18–30, and > 30° N) vs. three longitude (< − 82, − 82 to − 60, and > − 60 °W) groups plus three gross latitude groups independent of longitude and vice-versa. Among nearly 54,000 reported wind speeds, 80% measured less than the minimum (64 knots) for “hurricane” classification. Consequently, for analysis, hurricane track data reflected observation frequency rather than wind intensity^28^.

The best fit models for nests and hatchlings per nest hindcasted each metric to 1939, eight years prior to the 1947 reference point^10^ since age 8 reflects the youngest age of sexual maturity for this species^9^. For climate series that began later than 1939, respective mean values for the first “x” years (where “x = number of missing years”) conservatively populated the missing series data between 1939 and the series start year. To test if nest protection exacerbated hatchling production^35^, descriptive statistics compared predicted vs. observed nests and hatchlings per nest during 1966–1985, 1986–2005, and 2006–2022.

### Objective 3: constructing and validating a population simulator

Three life history equations fostered a feedback loop for building a population simulator:

(i) Nests = mature females x (clutch frequency divided by remigration interval);
(ii) Female hatchings = nests x hatchlings per nest x proportion female;
(iii) Mature females = female hatchlings x age-based survival schedule.

Not all sea turtle hatchlings originating in Mexico remain in the GOM^29^, and prior studies urge accounting for emigration in population modeling^30^. Extending climate reconstruction of particle emigration from the GOM into the Northwest Atlantic Ocean^16^ to 1950–2022 produced normally distributed residuals (*P* = 0.858). Given an apparent randomized effect and limited reports of Kemp’s ridleys leaving the Northwest Atlantic to nest in the GOM^30^, annual emigration proportion remained constant (0.84) during simulator runs.

Static value assignment across all life history variables comprised the null simulator model. A static value of 49.6 hatchlings per nest reflected 103.3 (i.e., the mean of annual mean clutch size values during 1966–1992^36^ less 1) multiplied by 0.6 hatching emergence, plus an additional assumption that 0.2 would not reach the ocean (i.e., a second multiplier of 0.8 would). A static female proportion value of 0.66 reflected a mid-range (2:1) female to male bias per prior Kemp’s ridley sea turtle studies^37^. Static sexual maturity at age 10 and a static clutch frequency to remigration interval of 1.25 matched prior modeling studies^9,14,15,21,22^.

The present study used the following static age-based survival that reflected the allometric intent of a Kemp’s ridley Stock Assessment Model^14^ but with lower survival rates due to prior study bias for population growth (Supplement S1): age 0 (A0) = 0.174; A1 = 0.318; A2 = 0.466; A3 = 0.548; A4 = 0.601; A5 = 0.655; A6 = 0.717; A7 = 0.779; A8 = 0.837; A9 = 0.880; A10 = 0.907; A11 = 0.921; ≥ A12 = 0.93). Spatial location did not influence survival rate, and most post-hatchlings transition from oceanic to GOM neritic habitats occurs before age 2^13^. This survival schedule also supported a sex-independent age structure that produced, per the inverse power equation, a survival equivalent score^16^ of 0.91 for the ratio of ages A10–A34 vs. ages A1–A9. This survival equivalent score mirrors the score for Kemp’s ridley sea turtles captured by coastal research trawl surveys in the Northwest Atlantic Ocean since 1990^16^.

Like static values, dynamic ranges for life history variables required standardization. Temporal variability in hatchlings per nest reflected climate-based prediction in Objective 1. All four remaining variables oscillated as a linear function of the maximum and minimum hindcasted nests between 1939 and 2022 (Objective 1). Age of sexual maturity for Kemp’s ridley sea turtles spans ± 20% age 10^9,14,15,21,22^; thus, female proportion and the ratio of clutch frequency to remigration interval also varied by ± 20%. Due to a maximum proportional increase of 0.065 for the highest survival rate (0.93) to reach 0.99, temporal variability in survival only spanned ± 3% of the static age-based survival schedule. Lastly, linear correspondence between dynamic life history values and annual nest counts necessitated pairing age of sexual maturity and female proportion with cohorts that originated 10 years prior to variable computation year to best align with the simulated nest year effect.

The population simulator projected 167 cohorts during 1856–2022, with the first cohort year set as the AMO series start year nearly a century prior to the 1947 nesting benchmark^10^. To prevent premature cohort disappearance in the simulator, sea turtles could attain a theoretical maximum age of 160 years old. This age corresponds to > 5 times the maximum contemporary age reported for this species in the GOM^13^, and > 50% older than a maximum age of 102^38^ suggested for a survival schedule strongly biased for population growth (Supplement S1). Static age-based survival also fostered an 1856 ‘burn-in’ assemblage that supported 30,000 nests annually, 20% greater than the de-listing criteria of 10,000 annual nesters at a constant clutch frequency to remigration interval ratio of 1.25^9^.

Systematic multiplication of annual age group abundance (integer) by the appropriate age-based survival rate simulated annual abundance for each cohort across years. Annual mature females reflected cohort survival to maturity, which for simplicity applied to all cohort survivors in the assigned maturation year. A post hoc annual addition of 452 nests ensured simulation of exactly 30,000 nests annually for the null (i.e., static life history) configuration. At 49.6 hatchlings per nest, the simulator computed 1.488 M combined-sex hatchlings annually, 45% more than peak hatchling production in 2009^9^. Among these, 824,947 female hatchlings (i.e., 0.66 proportion female × 0.84 retained in GOM) fed back into the simulator annually by enabling the “iterative function” to link hatchlings entering and produced by the simulator.

Hierarchical cluster analysis validated simulator performance across static (0) vs. dynamic (1) parameterization of life history variables using correlation strengths for regression-predicted (Objective 1) and simulator-predicted annual nest counts during 2006–2022. After substitution of observed hatchlings per nest during 1966–2022, a second cluster analysis re-evaluated correlation strengths between simulated and observed nest counts.

### Objective 4: simulating historical and contemporary assemblage dynamics

The historical Kemp’s ridley sea turtle nesting reference in Tamaulipas, Mexico represents an arribada event from 18 June 1947^10^. Rather than further speculate about the accuracy of 1947 nest estimates among sources^10–12^, this study emphasized reducing mature females to their presumed abundance in 1966 to best reconstruct annual nest count variability through 2022.

Two poaching weights (60%, 90%) at each of three 10-year increments prior to 1966 simulated a range of anthropogenic egg removal rates in Tamaulipas, Mexico since 1936^10^. With static values selected for all other life history variables, the slope of simulated nests with 60% annual poaching during 1946–1965 produced the closest slope (− 579) to the slope of observed nests (− 581) during 1966–1975. All six poaching weights produced nest count slopes of 40–150 during 1976–85, in contrast to a − 41 slope for observed nest counts during these years. Proportionate reduction (-0.094) of annual survival for ages ≥ 10 between 1946^36,39^ and 1965 simulated 5980 nests in 1966, just 11 fewer than the target nest count observed in the same year.

Additional edits reduced the gap between simulated and observed nest counts through 2022. First, observed hatchlings per nest replaced climate predicted hatchlings per nest. Subsequent cluster analysis with updated correlation strengths after this substitution refined life history variable associations with nests during 1966–2022. Next, dividing observed annual nest counts by the simulated mature female abundance in each year computed a coarse adjustment factor for the ratio of clutch frequency to remigration interval. Ultimately, selection of a single best life history variable configuration for further investigation reflected the least coarse adjustment to a 1.25 ratio of clutch frequency to remigration interval.

Coarse adjustments to the clutch frequency to remigration interval ratio produced a cascading impact on simulated abundance that required further but standardized correction protocols. Mexico did not fully implement trawling restrictions until 1979^15^; thus, when simulated nests exceeded observed nests prior to 1980, reduced survival of mature females in the year of nest count discrepancy achieved agreement between simulated and observed nests. Independent of year, when observed nests exceeded simulated nests, several steps achieved annual agreement. First, clutch frequency increased while remigration interval remained static. If the clutch frequency to remigration interval ratio reached 2.5, subsequent adjustments increased annual survival for ages ≥ 1 but only by ≤ 0.065. After reaching the upper limit of survival increase, clutch frequency adjustment resumed. Consequently, despite standardization, fine-scale simulation edits reflect a proxy rather than a precise pathway for achieving exact fit between simulated and observed nest counts during 1966–2022.

After resolving all discrepancies with observed nest counts for a single best simulation, a repeat run computed the reciprocal abundance for male Kemp’s ridley sea turtles. Consolidation of male and female simulations preceded calculation of six annual demographic metrics: (a) mixed-sex mature abundance; (b) mixed-sex immature abundance; (c) female proportion of the mature component; (d) female proportion of the immature component; (e) mature proportion of the overall assemblage; (f) neophyte proportion of mature females. Correlation identified associations between demographic metrics and simulated adjustment to the clutch frequency to remigration interval ratio.

## Results

### Objective 1: time series analysis

**Figure 1:**
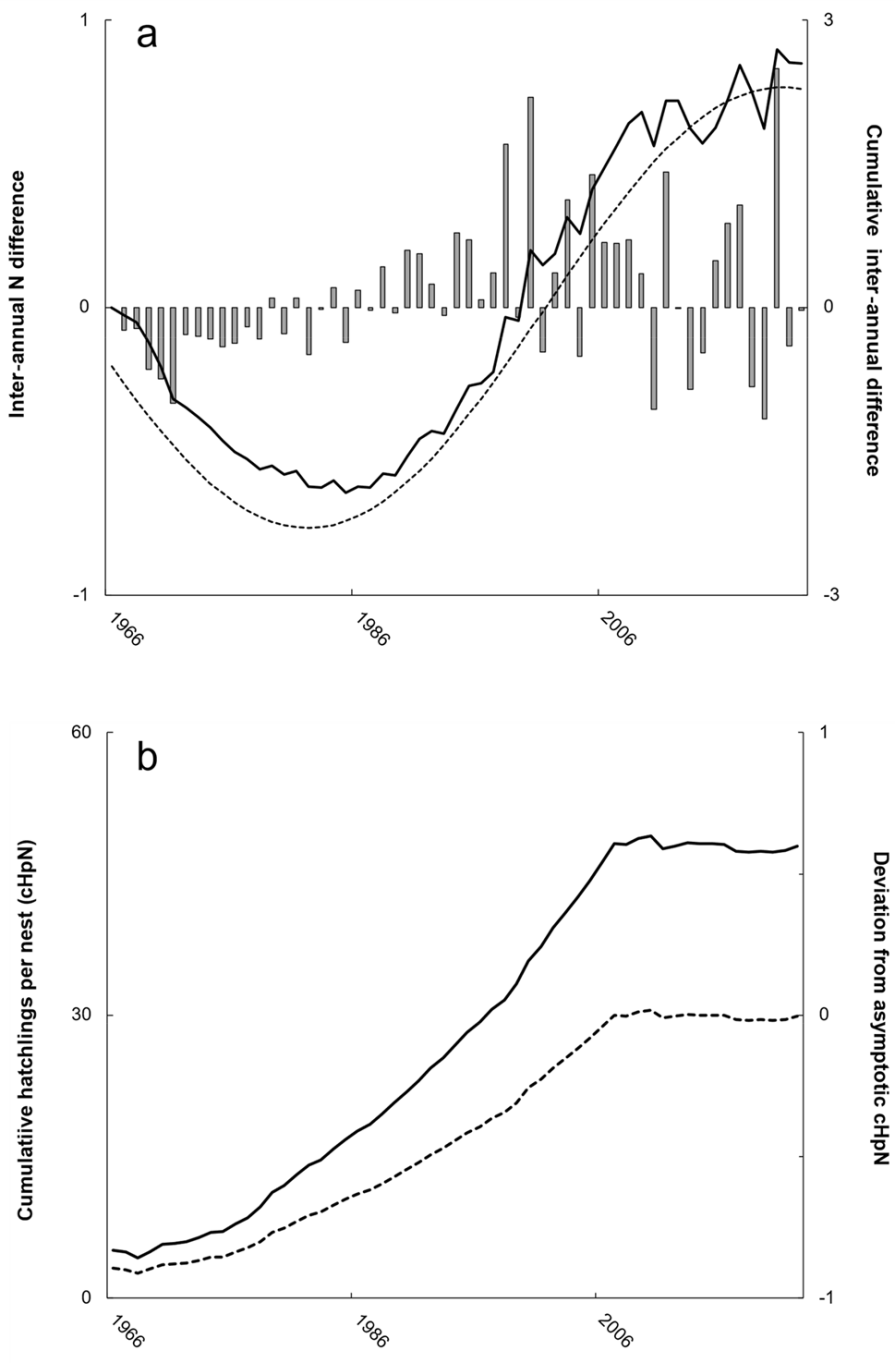
Critical metrics from analysis of Kemp’s ridley sea turtle nest counts (1966–2022). In (**a**), proportionate inter-annual differences (bars, first y-axis) summed to create a smooth cumulative series (solid black line, second y-axis) that best aligned with a periodicity- and amplitude-adjusted sine wave (black dashed line). In (**b**), cumulative hatchlings per nest (cHpN; solid line, first y-axis) and deviation from asymptotic cHpN (dashed line, second y-axis) systematically increased from 1968 through 2007.

During 1966–2022, median nests totaled 3369 but ranged from 702 (1985) to 22,415 (2017). Despite grand median inter-annual variation of − 1%, peak inter-annual fluctuation ranged from − 39% during 2018–2019 followed by 83% during 2019–2020 (Fig. 1a). Absolute magnitude of proportionate inter-annual change also significantly differed (H = 9.17, df = 2, *P* = 0.010), with increasing median values over time: 1966–1985 (10%), 1986–05 (15%), and 2006–2022 (27%).

Significant correlation (*P* < 0.001, r = 0.99) existed between the cumulative inter-annual nest count difference and a sine wave periodicity adjusted as observation year ÷ 12.25 (Fig. 1a). Sine wave minimum occurred in 1982, 3 years prior to nest count minimum. Sine wave maximum occurred in 2021, with a subsequent future minimum projected in 2059.

Temporal decline in the ratio of cumulative nests to 10-year lagged cumulative hatchlings resembled a power equation. Between 1986 (0.16) and 2014 (0.06), the annual ratio of these two metrics declined at a rate of 0.1548x^−0.267^ (r^2^ = 0.93), where “x” = sequential observation order. Expanding temporal coverage to 1976–2022 produced a steeper decline (1.0923x^−0.831^, r^2^ = 0.99) and value range (i.e., 1.08 in 1976, 0.04 in 2022).

Annual hatchlings per nest ranged from 5.1 (1966) to 80.7 (1989) which corresponded to a cumulative hatchling per nest range of 5.1 (1966) to 49.1 (2010). Cumulative hatchlings per nest reached an asymptote of 48.2 in 2007 then stabilized thereafter (Fig. 1b). The rate of achieving this asymptote increased temporally, with 24 years between furthest from asymptote (1968) and 50% discrepancy (1992) but only 15 additional years to reach asymptote (Fig. 1b).

### Objective 2: climate prediction of nesting metrics

**Figure 2:**
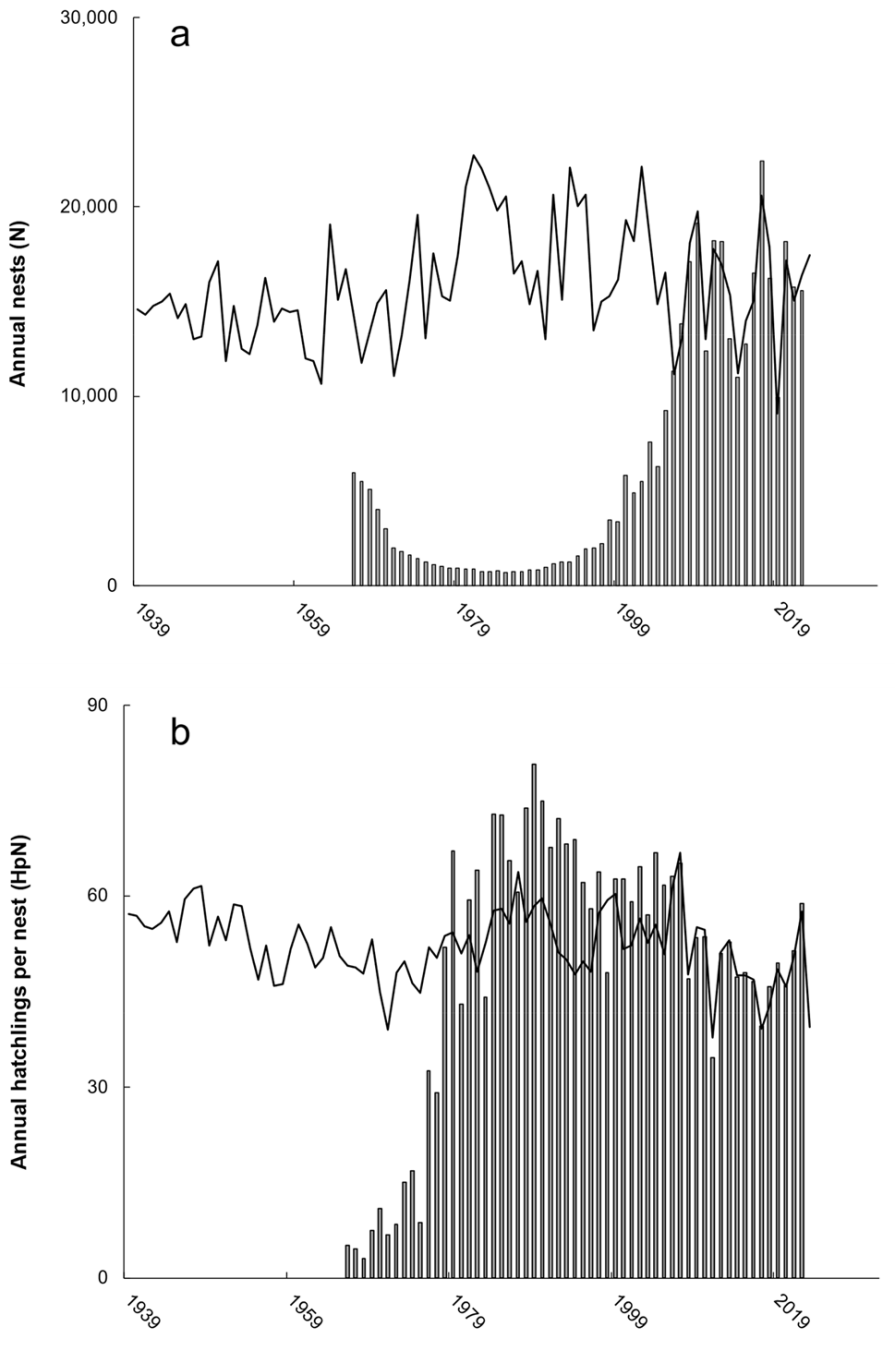
Observed (bars) vs. regression-predicted (black lines) annual (**a**) nests or (**b**) hatchlings per nest. For both metrics, hindcasting extended to 1939 to encompass eight years prior to the 1947 historical benchmark. For both metrics, forecasting only extended one year coinciding with the shortest lag interval association among climate series evaluated (see Table1).

Regression of best lagged climate associations predicted (*P* < 0.001, adj r^2^ = 0.83) nesting during 2006–2022 to within 29 nests and with normally distributed residuals (*P* = 0.712). Hindcasting this predictive equation to 1939 suggested that, had assemblage disruption not occurred, between 9,951 (2019) and 22,709 (1981) nests may have occurred annually since 1966 (Fig. 2a). Overall, observed nests comprised 39% of nests predicted from climate association, with similar discrepancy during 1966–1985 (− 294,889 nests) vs. 1986–2005 (− 284,269 nests).

Regression of best lagged climate associations predicted (*P* < 0.001, adj r^2^ = 0.94) hatchlings per nest (2006–2022) to within 4,313 hatchlings, with normally distributed residuals (*P* = 0.785). Hindcasting this predictive equation to 1939 suggested that, had assemblage disruption not occurred, between 37.8 (2011) and 66.9 (2007) hatchlings per nest may have emerged annually since 1966 (Fig. 2b). Observed hatchlings during 1966–2022 comprised 96% of predicted from climate association. During 1966–1985, predicted exceeded observed hatchlings by 3,877,951, but during 1986–2005 observed exceeded predicted hatchlings by 3,112,662.

**Figure 3:**
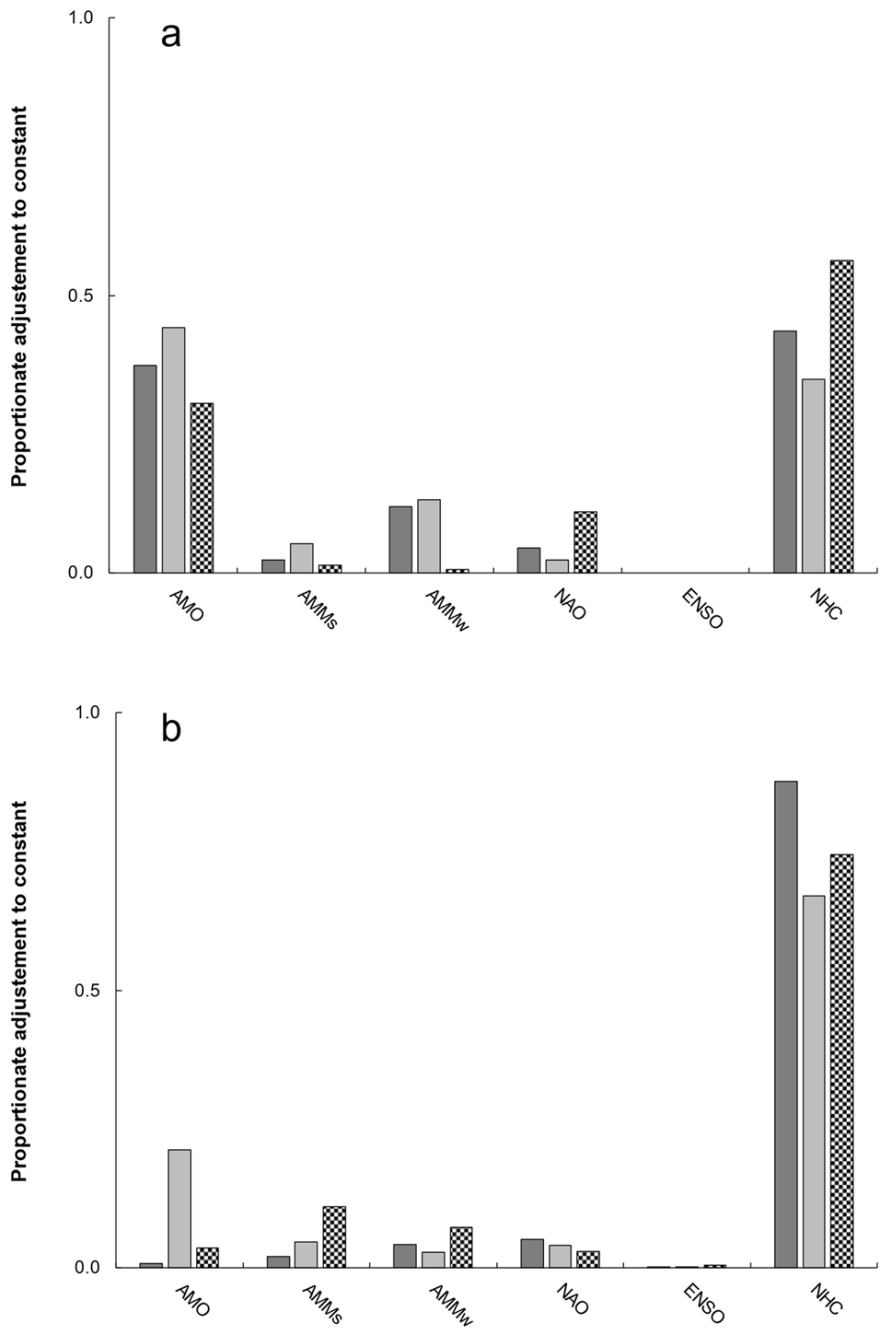
Gross adjustment to regression equation constants (**a** nests, **b** hatchlings per nest) by climate series (bars, x-axis) during 1966–1985 (dark gray), 1986–2005 (light gray), and 2006–2022 (pattern filled). Climate data series, represented along the x-axis, as follows: Atlantic Multidecadal Oscillation (AMO); Atlantic Meridional Mode, sea surface temperature (AMMs) and wind (AMMw); North Atlantic Oscillation (NAO); El Niño-Southern Oscillation (ENSO); National Hurricane Center (NHC), Atlantic hurricane database.

All climate data series except for the El Niño-Southern Oscillation (Table 1) predicted annual nest counts. The 9-year lag association with the AMO and 13-year lag association with cyclonic activity at lower latitudes in the eastern Atlantic Ocean collectively accounted for > 80% of annual proportionate adjustment to the regression equation constant (Fig. 3a).

**Table 1:** Climate association (r-value) and prediction (co-efficient, Co-E; *P* value) of Kemp’s ridley sea turtle nests (top panel) and hatchlings per nest (bottom panel) during 2006–2022.

Index	Nests		Co-E	*P* value
	Mo, Lag Yr	r-value		
AMO	Dec, L9	− 0.69	− 10,474.0	0.037
AMMs	Apr, L9	− 0.71	− 305.0	0.127
AMMw	Mar, L12	0.66	166.9	0.093
NAO	Aug, L1	− 0.64	− 580.0	0.239
ENSO	MAM, L9	− 0.65		
NHC	C1, L13	0.75	24.4	0.133
		Constant	14,321.0	< 0.001
			Model	< 0.001
			Adj. r-sq	0.83
			Mallows Cp	5.0

Index	Hatchlings per nest		Co-E	*P* value
	Mo, Lag Yr	r-value		
AMO	Mar, L13	− 0.89	− 15.5	0.017
AMMs	Oct, L13	− 0.80	− 0.7	0.059
AMMw	Jun, L2	0.68	0.5	0.065
NAO	Feb, L7	0.61	1.2	0.093
ENSO	DJF, L1	− 0.58	− 0.7	0.242
NHC	Prop C, L5	− 0.78	− 20.7	0.013
		Constant	60.3	< 0.001
			Model	< 0.001
			Adj. r-sq	0.94
			Mallows Cp	7.0

Six climate indices evaluated included the Atlantic Multidecadal Oscillation (AMO); sea surface temperature (AMMs) and wind (AMMw) components of the Atlantic Meridional Mode; the North Atlantic Oscillation (NAO); the El Niño-Southern Oscillation (ENSO); and the National Hurricane Center (NHC) Atlantic database.

All six climate data series predicted annual hatchlings per nest (Table 1). The 5-year lag association with cyclonic activity in the eastern Atlantic independent of latitude accounted for the single greatest (0.67–0.88) proportionate adjustment to the regression equation constant across time periods, with peak influence during 1966–1985 (Fig. 3b).

### Objective 3: constructing and validating a population simulator

Thirty-one (non-null) configurations of demographic and survival rates enabled pairwise examination of correlation strengths between (i) simulated and climate-predicted annual nest counts and between (ii) simulated and observed annual nest counts. Cluster analysis revealed ≥ 99% similarity between these two distributions during 2006–2022, which validated simulator functionality. Substituting observed values for climate-predicted values of hatchlings per nest marginally increased (i.e., 99 vs. 98% similarity) association between the clutch frequency to remigration interval ratio and simulated nests during 1985–2006. This substitution also improved association (i.e., from 77 to 88% similarity) between dynamic sexual maturity age and the annual slope of simulated nests independent of year. Lastly, substituting observed hatchlings per nest since 1966 altered survival association with simulated nests from 77% similarity with 1986–2005 to 50% similarity with 2006–2022.

### Objective 4: simulating historical and contemporary assemblage dynamics

Reducing simulator abundance via egg poaching and mature female pathways during 1946–1965 further refined cluster analysis associations with observed nest counts between 1966 and 2022. Dynamic clutch frequency to remigration interval ratio remained 99% similar with observed nest counts during 2006–2022, but also became the top demographic association (74% similarity) for observed nest counts during 1966–1985 and 1986–2005 (which also became 94% similar overall). Dynamic age of sexual maturity joined the slope of simulated annual nest counts from 1966 through 2022 with 87% similarity but exhibited the least (3% similarity) association with nests. Dynamic proportion female and survival exhibited 50% similarity with observed nests during 2006–2022 but only 19% similarity with nests observed between 1966 and 2005.

Gross (1966–2022) annual adjustment to the clutch frequency to remigration interval ratio ranged from 0.43 to 0.98, with the least adjustment associated with dynamic maturity age and dynamic female proportion. After implementing the coarse clutch frequency adjustment associated with this life history variable configuration, additional adjustments included (i) no survival decrease prior to 1980 and (ii) a cap on survival increase during 1998–2012. Following all adjustments, a net difference of zero existed between simulated and observed nests.

**Figure 4:**
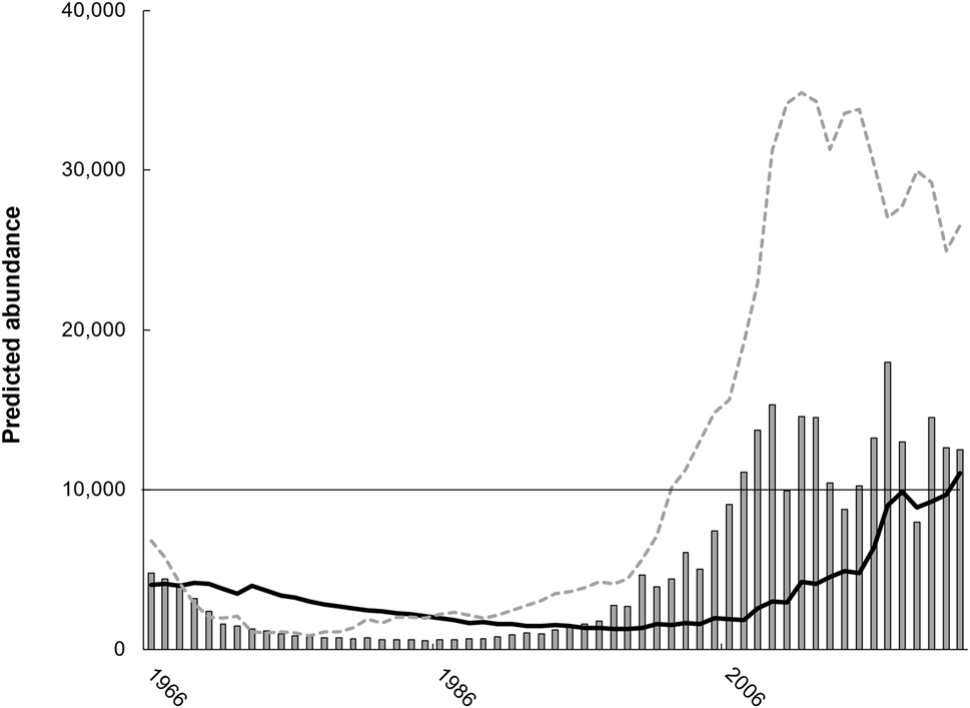
Estimated abundance of mature female Kemp’s ridley sea turtles since 1966 with (i) no change in annual reproductive activity (bars) vs. (ii) for the simulated assemblage (black line). Gray dashed line denotes immature female abundance in the same simulation. Thin black horizontal line denotes 10,000 mature females, a criterion for downlisting this species from endangered to threatened under the United States Endangered Species Act.

**Figure 5:**
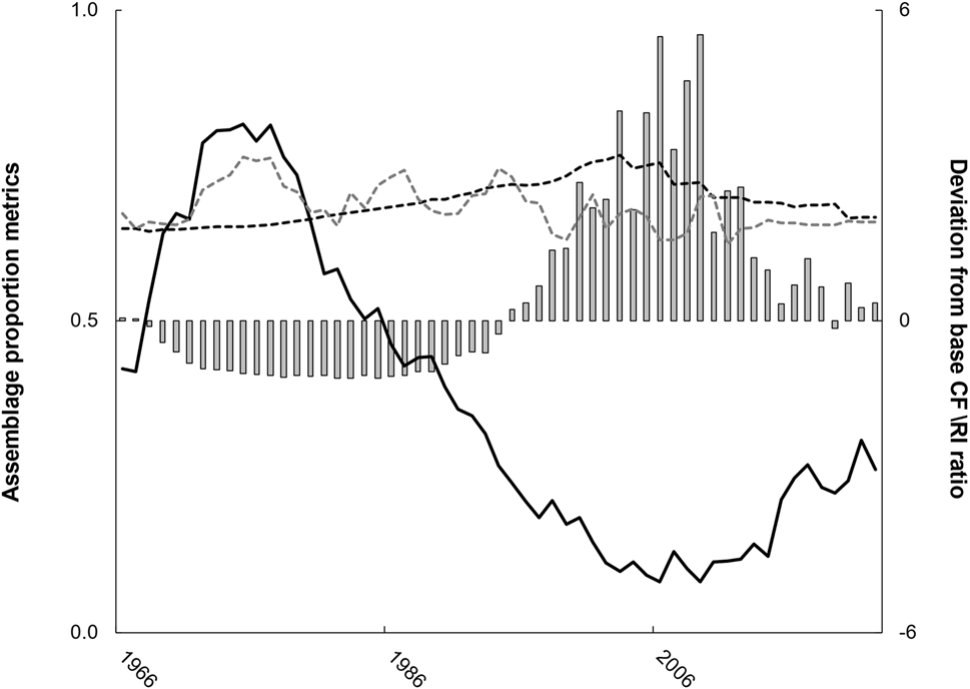
Demographic metric associations (lines, first y-axis) with adjustment to the clutch frequency (CF) to remigration interval (RI) ratio (bars, second y-axis) that simulated Kemp’s ridley sea turtle nest counts (1966–2022). Featured demographic metrics include mixed-sex mature assemblage component (solid black line); female proportion of the mature component (black dashed line); and female proportion of immature component (gray dashed line).

The simulated assemblage comprised 4,239 mature females in 1966, fewest mature females (914) in 1997, and the most mature females in 2021 (10,182; Fig. 4). Comparatively, had all nest count variability since 1966 reflected abundance changes, mature females would have totaled 4072 in 1966, with fewest (561) in 1985 and most (17,932) in 2017 (Fig. 4). From 1966 through 1992, the ratio of simulated immature to mature females remained < 2 but then rapidly increased through 2009 (11.0) before exhibiting a steady decline concurrent with a simulated increase in mature females (Fig. 4). Temporal shift in the ratio of simulated immature to mature female abundance represented the most significant (r = 0.93) association overall and with respect to deviation in the clutch frequency to remigration ratio (Table 2). Closely related, the combined-sex mature proportion of the simulated assemblage (Fig. 5) exhibited the strongest inverse correlation with temporal ratio deviation (r = − 0.78, Table 2).

**Table 2:** Significant (*P* < 0.05) and non-significant (–) correlation among demographic metrics in a simulated assemblage following annual adjustment to reproductive activity (CF/RI) and neritic survival to reconstruct nest counts during 1966–2022.

	CF-RI	IF/MF	Abund (M)	Abund (I)	PF (M)	PF (I)	Prop M
IF/MF	0.93						
Abund (M)	–	–					
Abund (I)	0.55	0.66	0.64				
PF (M)	0.73	0.76	− 0.44	–			
PF (I)	− 0.47	− 0.41	− 0.30	− 0.51	–		
Prop M	− 0.76	− 0.85	–	− 0.66	− 0.79	0.55	
Prop Neo	− 0.38	− 0.50	–	− 0.29	− 0.56	0.34	0.69

Row labels as follows: IF/MF = ratio of immature (I) to mature (M) females (F); Abund = combined sex abundance for the M or I assemblage components; PF = proportion female within the M or I assemblage component; Prop M = combined sex mature component; Prop Neo = neophyte proportion of mature females.

The simulated female proportion of the mature component peaked in 2003 (0.77) when the mixed-sex mature component comprised just 0.10 of neritic abundance (Fig. 5). However, when the mature component peaked at 0.31 of neritic abundance in 2021, females comprised a smaller proportion (0.67). Conversely, the female proportion of the immature component peaked at 0.76 in 1977 but totaled < 1,000 immature males and females combined. After 1977, the female proportion of immature Kemp’s ridley sea turtles steadily declined in an oscillatory manner through 2011 (Fig. 5). After 2011, the apex of simulated immature abundance (52,245), the simulated immature female proportion stabilized (Fig. 5) concurrent with annual decline in immature female abundance (slope = − 1159).

## Discussion

Several success stories for sea turtle nesting have emerged following decades of conservation, and improved Kemp’s ridley sea turtle nesting since 1966 merits high recognition among them. For at least two decades prior to initiating routine beach monitoring, reduced cohort recruitment occurred due to extensive predator disturbance plus intensive egg harvesting^10^. At minimum, among mature females, annual neophyte recruitment must offset remigrant attrition for stability. As such, even if 95% of mature females survived annually, yearly unmitigated loss of 5% would reduce their abundance by two-thirds after 20 years. Kemp’s ridley sea turtles typically reach sexual maturity within the first decade of life^13^. Consequently, after decades of reduced future recruitment, nesting continued to decline during the first decades of conservation^9^. Nevertheless, without high survival rates since 1966, Kemp’s ridley sea turtle nest counts may not have increased after the 1985 minimum as further elaborated upon herein.

Until now, two considerations clouded a more favorable perception of the Kemp’s ridley sea turtle nesting trend: (i) unintentional parameterization of models that biased for population growth (Supplement S1) and (ii) expecting a long period of exponential growth^18–20^. Regarding the first matter, population simulations should always feature a null model that eliminates statistical artefacts. Unfortunately, prior modeling studies did not report null models^9,14,15,21,22^ and extrapolation of their life history values computed more future than starting mature females (Supplement S1). Regarding the second matter, only density-dependence received consideration to explain the nesting trend after 2009^18–20^. Following correction of prior methodological issues, re-analysis suggested a power rather than logistic relationship between nests and time-lagged hatchlings (Fig. 1b). More informative, the non-time lagged ratio of cumulative hatchlings to cumulative nests remained asymptotic after 2006. Given similarity between asymptotic (48.2) and null model (49.6) values for this metric, systematic assemblage rebuilding better explains nesting trends after 2009 than density-dependence. Systematic rebuilding also coincided with an increased rate to achieve the asymptotic state after 1992. During this period of faster ascension to asymptote, simulator adjustments included biologically questionable increases in survival rates and the ratio of clutch frequency to remigration interval. Commencement of assemblage rebuilding earlier than became mechanistically necessary in the simulator also best explains these drastic simulation manipulations during 1998–2012.

Positive annual slope for cumulative hatchlings per nest through 2007 also undoubtedly contributed to decadal periodicity in cumulative inter-annual nest count differences (Fig. 1a). Prior time series analyses^18,20^ did not consider inter-annual differences in a cumulative sense, which as demonstrated herein (and elsewhere^32^), provides a unique and valuable perspective. Although inter-annual difference amplitude increased in later years, the cumulative distribution of these differences oscillated smoothly between nearly symmetrical upper and lower bounds that resembled a low frequency sine wave. Modest increase in the proportionate amplitude of inter-annual nest count differences since 1966 aligned with increased mature female abundance and/or a reduced clutch frequency to remigration interval ratio. Despite substantial potential ‘over-production’ of hatchlings during 1986–2005 (Objective 1), the rate of cumulative hatchlings to cumulative nests increased gradually. This finding suggests that cumulative hatchling per nest increase comprised a small but critical component of the long-term oscillation. Theoretical^40^ and actual data^41^ also report oscillatory population dynamics across taxa.

Landmark theoretical ecology studies also illuminate the importance of temporal lags^42^. In the present study, in addition to the cumulative hatchling per nest increase preceding annual nest count increase by decades, climate associations occurred 5–13 years prior to nesting year. In the Americas, several studies report decadal lag association between sea turtle nesting and the AMO^24–27^, plus shorter lag intervals with the North Atlantic Oscillation^25^ and non-indexed sea surface temperature^43^. Decadal (and multi-decadal) associations influence cohort demographics that in turn influence future nesting^26,27^. Shorter lag interval associations may reflect altered reproductive activity in years leading up to and including nesting^43,44^. Multiple regression retained most climate data series, but cyclonic activity in the Northeastern Atlantic Ocean and the AMO disproportionately altered equation constants (Fig. 3).

AMO influences north–south GOM circulation patterns^45^ which in turn promotes cascading trophic effects^46^. Given a nine-year lag association between AMO and nests, differential foraging opportunities may have contributed to variable age of sexual maturity. Across simulations, dynamic maturity age exerted greatest influence on annual nest count slope, which became most evident during efforts to precisely recreate the 1966–2022 nest count trend. This finding may indicate reduced somatic growth concurrent with greater caloric investment in reproductive development^47^. Such interpretation offers a more favorable take on reduced contemporary somatic growth rates reported across sea turtle species in this region^48–51^.

Peak AMO lag association with hatchlings per nest occurred at an even longer (13 years) lag interval than the lag AMO association with nests but mirrored a decadal lag association reported between AMO and green sea turtle clutch attributes in the southern GOM^27^. This association could represent a fitness advantage across cohorts that hatch in years when environmental conditions favor greater emergence and crawl success^52^. Alternatively, and/or in conjunction with, the 13-year lag association could indicate temporal variability in nest incubation conditions. Incubation conditions at Mexican nesting beaches vary with geomorphological processes and biological activity, notably past nesting events (see review in^53^).

Cyclonic activity within the GOM influences the spatial distribution of post-hatchling Kemp’s ridley sea turtles during the first six months of life^28^; however, here cyclonic associations occurred (i) outside of the GOM and (ii) 5–13 years prior to nesting. Despite western GOM origin, greatest associations occurred in the northeast Atlantic Ocean at (i) latitudes south of the GOM southern boundary for nests but (ii) independent of latitude for hatchlings per nest. Nest association may reflect temporal variability in *Sargassum* sp. abundance within the GOM^54^, which provides critical protective cover and developmental habitat for Kemp’s ridley sea turtles in oceanic habitats early in life^55^. Given a shorter lag interval association for hatchlings per nest, this association could reflect temporal variability in parental foraging opportunities. Specifically, if altered foraging impacted clutch frequency and/or remigration interval^23^, an underlying caloric basis for variability could also manifest as variable allocation of energy to eggs during embryonic development. Following coarse adjustment, reconstructing annual nest counts prior to 1985 predominantly required reducing the clutch frequency to remigration ratio. This period of lowest ratio also coincided with a sustained period of cooling in the GOM^45^ and may also help explain the perceived loss of ‘old nesters’ concurrent with the fewest annual nest counts in the 1980’s^36^.

In addition to climate-mediated foraging opportunities, changes in demographic structure, notably among mature females, could have also influenced their annual reproductive activity. Across sea turtle species, neophyte and remigrant mature females exhibit similar clutch frequency^56,57^. Some of this lack of discrepancy may reflect neophyte nesters only comprising a substantial proportion of the mature population during rebuilding periods^25,57^. In the present study, the simulated proportion of mature females tripled (0.1 to 0.3) between 2003 and 2021 (Fig. 5). This observation supports rapid rebuilding by an influx of neophyte nesters. However, despite decades of conservation the mature proportion never resumed levels simulated in the late 1970s (Fig. 5) following demographic disruption during 1946–1965. Consequently, the relative nest count plateau since 2009 also suggests further evidence of Kemp’s ridley sea turtles resuming a more stable age structure than existed in 1966 given that higher annual nest counts since 2005 occurred with a smaller mature female proportion.

Improved collection and analysis of demographic data could substantiate simulated trends. As noted in the Methods, the null model survival equivalent score of 0.91 corresponds to ages A10 through A34 comprising 0.2 of ages 1–34. As such, a smaller rather than larger^18^ mature proportion reflects demographic structure best aligned with the null model. Green sea turtle models also suggest improved breeding periodicity with nester abundance^58^, consistent with the dramatic simulated increases in survival and increased ratio of clutch frequency to remigration interval required to achieve nest counts after 2000. Temporal re-analysis of GOM in-water data sets should emphasize survival equivalence, which effectively scales catch rates for age structure^16^. Genetic fingerprinting^59^ of mature females on nesting beaches could also improve prediction of clutch frequency and remigration interval. To date, this technique remains under-utilized both at nesting beaches in Mexico^60^ and at the most critical nesting colony for Kemp’s ridley sea turtles in the United States^61^.

Compared to when conservation efforts began, age structure has vastly improved for Kemp’s ridley sea turtles in the GOM; however, as a precaution, several caveats warrant expression. First, strong climate associations suggest future temporal oscillation. Only time, measured in decades, will determine if future oscillation magnitudes more closely resemble the nesting time series since 2009 or the long-term oscillation since 1966. A trajectory resembling high frequency but low amplitude climate-mediated oscillations hindcasted in Objective 1 seems plausible. Second, hatchlings released since 2013 have not likely contributed much to nesting thus far. Post-2012 cohorts comprised fewer hatchlings than in the preceding decade, further suggesting a future downturn in nesting once those cohorts reach sexual maturity. Barring changes in demographic rates or survival, the null model nest and hatchlings per nest values predict nesting potential as Nests = [(30,000 * hatchlings]/(30,000 * 49.6)]. Accordingly, hatchlings released since 2013 may support 17,519 nests (range = 11,780 to 21,298) annually during 2023–2032. This suggestion emphasizes the final caveat: simulations that begin with a balanced null model deduce lower assemblage abundances^14^. As detailed in Objective 4, the simulated assemblage in 2022 comprised < 65,000 Kemp’s ridley sea turtles in the GOM across ages ≥ 1 and sexes. With 1/6th emigrating from the GOM annually (i.e., GOM retention = 0.84), only ~ 10,000 Kemp’s ridley sea turtles may exist in the Northwest Atlantic Ocean, far less than recently estimated without first also balancing a null model^16^. These conservative abundance estimates in turn provide crucial demographic context for appreciating the relative impact of and across annual mortality sources. Cold stun strandings and associated rehabilitation response^62,63^ plus reducing incidental take across a range of anthropogenic activities^10,64–66^ remain the greatest opportunities to positively impact this species by lessening annual mortality.

Kemp’s ridley sea turtle nesting may not achieve 1947 levels^10,12^ by 2048, but the initial demise and recovery ‘saga’ of this species remains far more ‘success’ than ‘setback’^20^. Following three decades of simulated anecdotal recruitment loss^10^, cumulative hatchlings per nest did not reach a suggested stable point until 2007, despite nearly 40 years of conservation. The historically prevalent arribada form of nesting may have also exacerbated mature female susceptibility to trawl capture^36,64^ due to extended adult mass aggregation in nearshore coastal waters^53^. Arribada and solitary nesting strategies each pose evolutionary pros and cons^53^, but given absence of historical level arribadas since 1966, solitary nesting can sustain this species. The 1947 arribada coincided with the apex of a long-term warming event^45^; thus, barring assemblage disruption, the present warm phase may have witnessed such a performance.

Conservation cannot control climate, but limiting anthropogenic sea turtle mortalities permits populations to navigate change to the best of their evolutionarily adapted capabilities. Climate change largely remains perceived as a “threat”^67^, but new momentum supports evaluating ecological time series data in the context of broad climate indices^68^. The ability “…to link, in time and space, climate-mediated dynamics across a wide range of species…^68^” embodies ecological thinking ripe for adoption by wildlife management. In closing, applying the analytical approaches presented herein to a myriad of taxa, sea turtle and otherwise, represents a vital first step towards the existential goal of balancing protections with optimized resource use.

### Supplementary Information

Supplementary Information.

## Data Availability

Following acceptance and publication, the datasets generated and analyzed for the present study will become available from the corresponding author upon reasonable request.

## References

[1] Caldwell DK (1963). The sea turtle fishery of Baja California, Mexico. Calif. Fish. Game.

[2] Wilson C, Tisdell C (2003). Conservation and Economic Benefits of wildlife-based marine tourism: Sea turtles and whales as case studies. Human Dimen. Wild..

[3] National Research Council. *Decline of the sea turtles: causes and prevention*. (National Academies Press, 1990).

[4] Andrew NL, Kennelly SJ, Broadhurst MK (1993). An application of the Morrison soft TED to the offshore prawn fishery in New South Wales Australia. Fish. Res..

[5] Lewison RL, Crowder LB, Wallace BP, Safina C (2014). Global patterns of marine mammal, seabird, and sea turtle bycatch reveal taxa-specific and cumulative megafauna hotspots. Proc. Nat. Acad. Sci..

[6] Balazs GH, Chaloupka M (2004). Thirty-year recovery trend in the once depleted Hawaiian green sea turtle stock. Biol. Conserv..

[7] Troëng S, Rankin E (2005). Long-term conservation efforts contribute to positive green turtle *Chelonia mydas* nesting trend at Tortuguero. Costa Rica. Biol. Conserv..

[8] Ceriani SA, Casale P, Brost M, Leone EH, Witherington BE (2019). Conservation implications of sea turtle nesting trends: elusive recovery of a globally important loggerhead population. Ecosphere.

[9] National Marine Fisheries Service and U.S. Fish and Wildlife Service. Kemp’s ridley sea turtle (*Lepidochelys kempii*) 5-year review: Summary and Evaluation, 1–62 (2015).

[10] Hildebrand, H. H. Hallazgo del área de anidación de la tortuga marina, “lora”, *Lepidochelys kempi* (Garman) en la costa occidental del Golfo de México. *Ciencia Méx*. **22**, 105–112 (1963). Translated to English by Caillouet, C. W., Jr. (2010).

[11] Dickerson, V. L. & Dickerson, D. D. Analysis of arribada in 1947 film at Rancho Nuevo, Mexico in *Proceedings of the 26th Annual Symposium on Sea Turtle Biology and Conservation* (compilers Frick M., A. Panagopoulou, A. F. Rees & K. Williams) 290–291 (International Sea Turtle Society, 2006).

[12] Bevan E, Wibbels T, Najera BMZ, Sarti L, Martinez FI (2016). Estimating the historic size and current status of the Kemp's ridley sea turtle (*Lepidochelys kempii*) population. Ecosphere.

[13] Avens L, Ramirez MD, Hall AG, Snover ML, Haas HL (2020). Regional differences in Kemp’s ridley sea turtle growth trajectories and expected age at maturation. Mar. Ecol. Prog. Ser..

[14] Gallaway BJ, Gazey WJ, Caillouet CW, Plotkin PT (2016). Development of Kemp’s ridley sea turtle stock assessment model. Gulf Mex. Sci..

[15] Heppell, S. S., Burchfield, P. M., & Peña, L. J. Kemp’s ridley recovery: How far have we come, and where are we headed? in *Biology and Conservation of Ridley Sea Turtles* (ed. Plotkin, P. T.) 325–335 (Hopkins University Press, 2007).

[16] Arendt MD, Webster RP, Schwenter JA (2022). High annual survival suggested by size structure of Kemp’s ridley sea turtles captured by coastal research trawling in the Northwest Atlantic Ocean since 1990. Endang. Species Res..

[17] Babcock, E. A., Barnette, M., Bohnsack, J., Isely, J. J., & Porch, C., *et al.* Integrated Bayesian models to estimate bycatch of sea turtles in the Gulf of Mexico and southeastern U.S. Atlantic coast shrimp otter trawl fishery. NOAA Tech Memorandum NMFS-SEFSC-721, 47 p. (2011).

[18] Caillouet CW, Raborn SW, Shaver DJ, Putman NF, Gallaway BJ (2018). Did declining carrying capacity for the Kemp’s ridley sea turtle population within the Gulf of Mexico contribute to the nesting setback in 2010–2017?. Chel. Conserv. Biol..

[19] Caillouet CW (2014). Interruption of the Kemp’s ridley population’s pre-2010 exponential growth in the Gulf of Mexico and its aftermath: One hypothesis. Mar. Turtle News..

[20] Caillouet, C. W. Jr., B. J. Gallaway & N. F. Putman. Kemp's ridley sea turtle saga and setback: Novel analyses of cumulative hatchlings released and time-lagged annual nests in Tamaulipas, Mexico. *Chelon. Conserv. Biol.* **15**, 115–131 (2016).

[21] Turtle Expert Working Group. An assessment of the Kemp’s ridley (*Lepidochelys kempii*) and loggerhead (*Caretta caretta*) sea turtle populations in the western North Atlantic. NOAA Tech Memorandum NMFS-SEFSC-409, 96 p. (1998).

[22] Heppell SS, Crouse DT, Crowder LB, Epperly SP, Gabriel W (2004). A population model to estimate recovery time, population size, and management impacts on Kemp’s ridley sea turtles. Chelon. Conserv. Biol..

[23] Kocmoud AR, Wang H-H, Grant WE, Gallaway BJ (2019). Population dynamics of the endangered Kemp’s ridley sea turtle following the 2010 oil spill in the Gulf of Mexico: Simulation of potential cause-effect relationships. Ecol. Model..

[24] Van Houtan KS, Halley JM (2011). Long-term climate forcing in loggerhead sea turtle nesting. PLoS ONE.

[25] Arendt MD, Schwenter JA, Witherington BE, Meylan AB, Saba VS (2013). Historical versus contemporary climate forcing on the annual nesting variability of loggerhead sea turtles in the Northwest Atlantic Ocean. PLoS ONE.

[26] Arendt MD, Schwenter JA, Owens WD, Valverde RA (2021). Theoretical modeling and neritic monitoring of loggerhead *Caretta caretta* [Linnaeus, 1758] sea turtle sex ratio in the southeast United States do not substantiate fears of a male-limited population. Global Change Biol..

[27] del Monte-Luna P, Nakamura M, Guzmán-Hernández V, Cuevas E, López-Castro MC, Arreguin-Sánchez F (2023). Multidecadal fluctuations in green turtle hatchling production related to climate variability. Sci. Rep.-UK.

[28] DuBois MJ, Putman NF, Piacenza SE (2020). Hurricane frequency and intensity may decrease dispersal of Kemp’s ridley sea turtle hatchlings in the Gulf of Mexico. Front. Mar. Sci..

[29] Putman NF, Mansfield KL, He R, Shaver DJ, Verley P (2013). Predicting the distribution of oceanic-stage Kemp’s ridley sea turtles. Biol. Lett..

[30] Caillouet, C. W. Jr., & Gallaway, B. J. Kemp’s ridley sea turtle emigration and immigration between the Gulf of Mexico and North Atlantic Ocean should not be ignored in age-structured population modeling. *Mar. Turtle News.* **161**, 9–14 (2020).

[31] Board, Ocean Studies, and National Research Council. Assessment of sea-turtle status and trends: integrating demography and abundance, 1–162 (National Academies Press, 2010).

[32] Manly BF, Mackenzie D (2000). A cumulative sum type of method for environmental monitoring. Environmetrics.

[33] Zuccaro C (1992). Mallows’ Cp statistic and model selection in multiple linear regression. Int. J. Market Res..

[34] Tran HD, Muttil N, Perera BJC (2015). Selection of significant input variables for time series forecasting. Environ. Modell. Softw..

[35] Caillouet CW (2021). Substantial reduction in annual production of Kemp’s ridley sea turtle hatchlings on beaches of Tamaulipas, Mexico may allow abundance of adults to increase. Mar. Turtle News..

[36] Márquez, R. Synopsis of biological data on the Kemp's ridley turtle*, Lepidochelys kempi *(Garman, 1880) Vol. 152. US Department of Commerce, National Oceanic and Atmospheric Administration, National Marine Fisheries Service, Southeast Fisheries Science Center (1994).

[37] Wibbels, T. Sex determination and sex ratio in ridley turtles. in *Biology and Conservation of Ridley Sea Turtles* (ed. Plotkin, P. T.) 167–189 (Hopkins University Press, 2007).

[38] Gallaway, B. J., Caillouet, C. W., Jr., Plotkin, P. T., Gazey, W. J., Cole, J. G., & Raborn, S. W. Kemp’s ridley stock assessment project. *Final report to Gulf States Marine Fisheries Commission*, 61 p. plus appendices (2013).

[39] Condrey, R. & Fuller, D. The U.S. Gulf Shrimp Fishery in *Climate variability, climate change* and fisheries (ed. Glantz, M. H.) 89–119 (Cambridge University Press, 1992).

[40] May RM (1974). Biological populations with nonoverlapping populations: Stable points, stable cycles, and chaos. Science.

[41] Turchin P, Taylor AD (1992). Complex dynamics in ecological time series. Ecology.

[42] Turchin P (1990). Rarity of density dependence or population regulation with lags?. Nature.

[43] Solow AR, Bjorndal KA, Bolten AB (2002). Annual variation in nesting numbers of marine turtles: The effect of sea surface temperature on re-migration intervals. Ecol. Lett..

[44] Arendt, M. D. Assessment of the probability of loggerhead sea turtle (*Caretta caretta*) recovery in the Northwest Atlantic Ocean within 50 years of federal and state protection in the US. Dissertation, 128 p. (University of South Carolina, 2016).

[45] del Monte-Luna P, Villalobos H, Arreguín-Sánchez F (2015). Variability of sea surface temperature in the southwestern Gulf of Mexico. Cont. Shelf Res..

[46] Karnauskas M, Schirripa MJ, Craig JK, Cook GS, Kelbe CR (2015). Evidence of climate-driven ecosystem reorganization in the Gulf of Mexico. Global Change Biol..

[47] Lester NP, Shuter BJ, Abrams PA (2004). Interpreting the von Bertalanffy model of somatic growth in fishes: The cost of reproduction. Proc. R. Soc. Lond. B..

[48] Bjorndal KA, Schroeder BA, Foley AM, Witherington BE, Bresette M (2013). Temporal, spatial, and body size effects on growth rates of loggerhead sea turtles (*Caretta caretta*) in the Northwest Atlantic. Mar. Biol..

[49] Bjorndal KA, Chaloupka M, Saba VS, Diez CE, van Dam RP (2016). Somatic growth dynamics of West Atlantic hawksbill sea turtles: A spatio-temporal perspective. Ecosphere.

[50] Bjorndal KA, Bolten AB, Chaloupka M, Saba VS, Bellini C (2017). Ecological regime shift drives declining growth rates of sea turtles throughout the West Atlantic. Global Change Biol..

[51] Ramirez MD, Avens L, Goshe LR, Snover ML, Cook M (2020). Regional environmental drivers of Kemp’s ridley sea turtle somatic growth variation. Mar. Biol..

[52] Fisher LR, Godfrey MH, Owens DW (2014). Incubation temperature effects on hatchling performance in the loggerhead sea turtle (*Caretta caretta*). PLoS ONE.

[53] Bernardo, J. & Plotkin, P. T. An evolutionary perspective on the Arribada phenomenon and reproductive behavioral polymorphism of olive ridley sea turtles (*Lepidochelys olivacea*) in *Biology and Conservation of Ridley Sea Turtles* (ed. Plotkin, P. T.) 59–87 (Hopkins University Press, 2007).

[54] Sanchez-Rubio G, Perry H, Franks JS, Johnson DR (2018). Occurrence of pelagic Sargassum in waters of the US Gulf of Mexico in response to weather-related hydrographic regimes associated with decadal and interannual variability in global climate. Fish. Bull..

[55] Witherington B, Hirama S, Hardy R (2012). Young sea turtles of the pelagic Sargassum-dominated drift community: Habitat use, population density, and threats. Mar. Ecol. Prog. Ser..

[56] Kendall WL, Stapleton S, White GC, Richardson JI, Pearson KN (2019). A multistate open robust design: population dynamics, reproductive effort, and phenology of sea turtles from tagging data. Ecol. Mon..

[57] Stokes KL, Fuller WJ, Glen F, Godley BJ, Hodgson DJ (2014). Detecting green shoots of recovery: The importance of long-term individual-based monitoring of marine turtles. Anim. Conserv..

[58] Piacenza SE, Balazs GH, Hargrove SK, Richards PM, Heppell SS (2016). Trends and variability in demographic indicators of a recovering population of green sea turtles *Chelonia mydas*. Endang. Species Res..

[59] Shamblin BM, Dodd MG, Griffin DB, Pate SM, Godfrey MH (2017). Improved female abundance and reproductive parameter estimates through subpopulation-scale genetic capture-recapture of loggerhead turtles. Mar. Biol..

[60] Camacho-Sanchez, F. Y., A. Alonso Aguirre, H. H. Acosta-Sánchez, H. Rodriguez-González, M. López-Hernández et al. DNA barcoding of Kemp’s ridley (*Lepidochelys kempii*) in Mexico. *First International Electronic Conference on Biological Diversity, Ecology, and Evolution*. 10.3390/BDEE2021-09392 (2021).

[61] Frey A, Dutton PH, Shaver DJ, Shelby Walker J, Rubio C (2014). Kemp’s ridley *Lepidochelys kempii* nesting abundance in Texas, USA: A novel approach using genetics to improve population census. Endang. Species Res..

[62] Griffin LP, Griffin CR, Finn JT, Prescott RL, Faherty M (2019). Warming seas increase cold-stunning events for Kemp’s ridley sea turtles in the northwest Atlantic. PLoS ONE.

[63] Caillouet CW, Putman NF, Shaver DJ, Valverde RA, Seney EE (2016). A call for evaluation of the contribution made by rescue, resuscitation, rehabilitation, and release translocations to Kemp’s ridley sea turtle (*Lepidochelys kempii*) population recovery. Herpetol. Conserv. Biol..

[64] Pritchard, P. C. H. & R. M. Márquez. Kemp’s ridley turtle or Atlantic ridley. IUCN Monograph No 2, Marine Turtle Series, 30 p. (1973).

[65] Putman NF, Hawkins J, Gallaway BJ (2020). Managing fisheries in a world with more sea turtles. Proc. R. Soc. Lond. B..

[66] Gallaway BJ, Gazey WJ, Wibbels T, Bevan E, Shaver DJ (2016). Evaluation of the status of the Kemp’s ridley sea turtle after the 2010 Deepwater Horizon Oil Spill. Gulf Mex. Sci..

[67] McCarty JP (2001). Ecological consequences of recent climate change. Conserv. Biol..

[68] Forchhammer MC, Post E (2004). Using large-scale climate indices in climate change ecology studies. Pop. Ecol..

