# Supplementary material for: Climate-mediated population dynamics for the world’s most endangered sea turtle species
Source: Sci Rep. 2023 Sep 2;13:14444. doi: 10.1038/s41598-023-41647-8 (PMC10475092; doi:10.1038/s41598-023-41647-8)
Supplement: Supplementary file 1 — Supplementary Information. [file 41598_2023_41647_MOESM1_ESM.docx]

**Supplement 1** for “Climate-mediated population dynamics for the world’s most endangered sea turtle species.”

As implied by “population dynamics”, the age structure of populations fluctuates temporally, and presumably oscillation occurs around a central density favoring stability [A]. As such, when modeling population responses to life history parameter changes, (a) ensuring that baseline life history equations are configured for stability is as important as (b) parameterizing life history metrics with biologically sound values. With this caveat in mind, gross synthesis was completed to evaluate prior modeling efforts for Kemp’s ridley sea turtle (KRST) population dynamics.

The basic equation for annual nesting in all sea turtle species is as follows:

Annual nests (N) = [Mature Females (MF) / Remigration Interval (RI)] x Clutch Frequency (CF)

This equation can be further simplified to N = MF x [CF/RI].

Annual MF reflects recruitment into the reproductive component of the population (neophyte), which must offset annual loss of remigrant MF to maintain a stable abundance of MF over time.

At large remigrant MF population levels, annual attrition magnitude can be substantial even with high annual survival rates [B]. For example, in 1947, the number of nesting KRST was estimated to be 48,607 (range = 33,006 to 83,981; C). Doubling these estimates to reflect a remigration interval of two years suggest existence of 66,012 to 167,962 MF KRST in 1947. Therefore, if 95% survived annually, between 3300 and 8398 neophyte MF KRST would need to recruit into the remigrant population annually to offset loss of MF KRST at this high survival. Coincidentally, these estimates of neophyte recruitment are on order with the upper end (5k) of annual loss estimates for all aged KRST from shrimp trawling before Turtle Excluder Devices (TEDs) were mandated, and several times greater than the low annual loss estimates (500; [D]).

Annual neophyte recruitment into the MF component of the population reflects several life history parameter considerations within at least a cohort, and potentially across multiple cohorts. Critical life history considerations include the number of hatchlings per nest (HpN) that enter the ocean, annual survival until reaching ASM, and the proportion of survivors to ASM that are female (Prop F). Temporal stability in MF does not guarantee lack of variability in annual N because CF/RI may also not remain static. Likewise, temporal variability in HpN and/or Prop F would also require compensatory life history adjustments to foster only population stability.

Three prominent population assessments have been conducted for Kemp’s ridley sea turtles, and most have used similar values for CF/RI (1.25) and female hatchling proportion (0.7; Table S1). Three variable but fixed (8, 10, 12) values for ASM were used by two modeling efforts [E,F,G], while the third modeled a mean ASM of 13.5 years computed from proportional weighting between A10 (0.1) and A14 (1.0) [I,J]. Fixed annual HpN of 48 [I,J] and 70 [E] were used by two modeling efforts, while the third used a mean HpN of 49 [F,G]. Extrapolation of these parameterization schedules across reported survival rates reveals that prior KRST population modeling produced more ‘replacement females’ than ‘starting females’. Consequently, all prior efforts biased for positive temporal slope, which necessitated reduced survival to fit true N data.

**Table S1**. Parameterization and slope ratio (*R*) for prominent Kemp’s ridley modeling to date.

^A^Turchin, P. “Population Regulation: Old Arguments and a New Synthesis*.” Population dynamics: new approaches and synthesis* (1995): 19-40.

^B^Arendt, M. D., J. A. Schwenter, B. E. Witherington, A. B. Meylan, and V. S. Saba. "Historical versus contemporary climate forcing on the annual nesting variability of loggerhead sea turtles in the Northwest Atlantic Ocean." *PLoS One* 8.12 (2013): e81097.

^C^Bevan, E., T. Wibbels, B. M Z. Najera, L. Sarti, F. I. Martinez, et al. "Estimating the historic size and current status of the Kemp's ridley sea turtle (*Lepidochelys kempii*) population." *Ecosphere* 7.3 (2016): e01244.

^D^National Research Council. “Decline of the sea turtles: causes and prevention.” National Academies Press, 1990.

^E^Turtle Expert Working Group. “An assessment of the Kemp’s ridley (*Lepidochelys kempii*) and loggerhead (*Caretta caretta*) sea turtle populations in the western North Atlantic.” NOAA Technical Memorandum NMFS-SEFSC-409 (1998), 96 p.

^F^Heppell, S. S., D. T. Crouse, L. B. Crowder, S. P. Epperly, W. Gabriel, et al. “A population model to estimate recovery time, population size, and management impacts on Kemp’s ridley sea turtles.” *Chelonian Conservation and Biology* 4.4 (2004): 765-771.

^G^National Marine Fisheries Service and U.S. Fish and Wildlife Service. “Kemp’s ridley sea turtle (Lepidochelys kempii) 5-year review: Summary and Evaluation.” (2015), 62 pages.

^H^Gallaway, B. J., C. W. Caillouet, Jr., P. T. Plotkin, W. J. Gazey, J. G. Cole, and S. W. Raborn. “Kemp’s ridley stock assessment project.” Final report to Gulf States Marine Fisheries Commission (2013), 61 p. plus appendices.

^I^Gallaway, B. J., W. J. Gazey, C. W. Caillouet, Jr., P. T. Plotkin, F. Alberto Abreu Grobois, et al. “Development of Kemp’s ridley sea turtle stock assessment model.” *Gulf of Mexico Science* 33.2 (2016): 138-157.
